# Physical Fitness and Telomere Length in Patients with Coronary Heart Disease: Findings from the Heart and Soul Study

**DOI:** 10.1371/journal.pone.0026983

**Published:** 2011-11-09

**Authors:** Jeffrey Krauss, Ramin Farzaneh-Far, Eli Puterman, Beeya Na, Jue Lin, Elissa Epel, Elizabeth Blackburn, Mary A. Whooley

**Affiliations:** 1 School of Medicine, University of California San Francisco, San Francisco, California, United States of America; 2 Division of Cardiology, University of Texas Southwestern Medical Center, Dallas, Texas, United States of America; 3 Department of Psychiatry, University of California San Francisco, San Francisco, California, United States of America; 4 Section of General Internal Medicine, Veterans Affairs Medical Center, San Francisco, California, United States of America; 5 Department of Biochemistry and Biophysics, University of California San Francisco, San Francisco, California, United States of America; 6 Department of Medicine, University of California San Francisco, San Francisco, California, United States of America; 7 Department of Epidemiology and Biostatistics, University of California San Francisco, San Francisco, California, United States of America; University of Valencia, Spain

## Abstract

**Background:**

Short telomere length (TL) is an independent predictor of mortality in patients with coronary heart disease (CHD). However, the relationship between physical fitness and TL has not been explored in these patients.

**Methods:**

In a cross sectional study of 944 outpatients with stable CHD, we performed exercise treadmill testing, assessed self-reported physical activity, and measured leukocyte TL using a quantitative PCR assay. We used generalized linear models to calculate mean TL (T/S ratio), and logistic regression models to compare the proportion of patients with short TL (defined as the lowest quartile), among participants with low, medium and high physical fitness, based on metabolic equivalent tasks achieved (METs).

**Results:**

229 participants had low physical fitness (<5 METS), 334 had moderate physical fitness (5–7 METS), and 381 had high physical fitness (>7 METS). Mean ± T/S ratio ranged from 0.86±0.21 (5349±3781 base pairs) in those with low physical fitness to 0.95±0.23 (5566±3829 base pairs) in those with high physical fitness (p<.001). This association remained strong after adjustment for numerous patient characteristics, including measures of cardiac disease severity and physical inactivity (p = 0.005). Compared with participants with high physical fitness, those with low physical fitness had 2-fold greater odds of having TL in the lowest quartile (OR 2.39, 95% CI 1.60–3.55; p<.001). This association was similar after multivariable adjustment (OR 1.94, 95%CI, 1.18–3.20; p = 0.009). Self-reported physical inactivity was associated with shorter TL in unadjusted analyses, but not after multivariable adjustment.

**Conclusions:**

We found that worse objectively-assessed physical fitness is associated with shorter leukocyte telomere length in patients with CHD. The clinical implications of this association deserve further study.

## Introduction

Telomeres are tandem repeat DNA sequences (TTAGGG) and associated proteins which form the protective caps at the ends of eukaryotic chromosomes [1], [2], [3]. Lack of maintenance of telomere length leads eventually to inability of cells to divide, and can cause genomic instability. Shorter telomere length is a marker of biological aging and can increase susceptibility to age-related diseases [4]. Short telomeres are associated with a variety of cardiovascular disorders, including atherosclerosis [5], [6], [7], congestive heart failure [8], left ventricular dysfunction [9], myocardial infarction [10], [11], [12], hypertension [13], [14], abdominal aortic aneurysm [15], and cardiovascular mortality [16], [17]. Given this consistent pattern of relationships, it is important to identify modifiable lifestyle factors that might promote telomere length maintenance.

Some studies suggest that self-reported physical activity is associated with longer leukocyte telomere length among healthy individuals [18], [19], [20]. However, only one small case-control study has examined the association between an objective measure of physical fitness (as opposed to self-reported physical activity) and telomere length. This study found that 30 sedentary patients had shorter telomere length and lower exercise capacity (measured by VO_2_max) than 27 endurance-trained adults [21]. To our knowledge, no other study has evaluated the association between an objective measure of physical fitness and telomere length, nor directly assessed whether subjective or objective measures of fitness are more strongly associated with telomere length. Therefore, we sought to evaluate the association between self-reported physical activity, treadmill exercise capacity and telomere length in 944 outpatients with stable coronary artery disease.

## Methods

### Participants

The Heart and Soul Study is a prospective cohort study investigating the influence of psychosocial factors on cardiovascular events in patients with stable coronary artery disease. The enrollment process has been previously described [22]. Eligible participants were recruited from outpatient clinics in the San Francisco Bay Area if they met at least one of the following inclusion criteria: 1) history of myocardial infarction, 2) angiographic evidence of at least 50% stenosis by area in at least one coronary artery, 3) evidence of exercise-induced ischemia by treadmill electrocardiogram or stress nuclear perfusion imaging, or 4) history of coronary revascularization. Individuals were excluded if they had a history of myocardial infarction in the past 6 months, deemed themselves unable to walk 1 block, or if they were planning to move out of the local area within 3 years.

The study protocol was approved by: the University of California San Francisco Committee on Human Research, the Research and Development Committee at the San Francisco VA Medical Center, the Medical Human Subjects Committee at Stanford University, the Human Subjects Committee at the VA Palo Alto Health Care System, and the Data governance Board of the Community Health Network of San Francisco. All participants provided written informed consent. Between September 2000 and December 2002, a total of 1024 participants enrolled in the study. Of these, 944 provided DNA samples and completed exercise treadmill testing.

### Telomere Length Assay

Genomic DNA was isolated according to standard procedures from peripheral blood leukocytes and stored at −70°C. Purified DNA samples were diluted in 96-well microtiter source plates to a fixed concentration of 3 ng/µL. Relative mean telomere length was measured from DNA by a quantitative polymerase chain reaction (qPCR) assay that compares mean telomere repeat sequence copy number (T) to a reference single-copy gene copy number (S) in each sample, as previously described and validated by comparison with Southern blot terminal restriction fragment analysis [23]. Standard curves were derived from serially diluted reference DNA as previously described and validated [23], [24], [25]. The T/S ratio was determined from the mean quantity of reference DNA found to match with each experimental sample for the copy number of the targeted templates (the number of telomere repeats for T and the number of beta-globin gene copies for S).

The primers for the telomere qPCR were tel1b (5′-CGGTTT[GTTTGG] 5GTT-3′) and tel2b (5′-GGCTTG [CCTTAC]5CCT-3′), used at final concentrations of 100 nM and 900 nM respectively. Human beta-globin gene qPCR primers were hbg1 (5′-GCTTCTGACACAACTGT-GTTCACTAGC-3′), used at a final concentration of 300 nM, and hbg2 (5′-CACCAACTTCATCCACGTTCACC-3′), used at a final concentration of 700 nM. All PCRs were carried out on a Roche Lightcycler 480 real-time PCR machine (Roche Applied Science, Indianapolis, Indiana).

To control for inter-assay variability, eight control DNA samples were included in each run. The T/S ratio of each control DNA was divided by the average T/S for the same DNA from each run to obtain a normalizing factor. The average normalizing factor across all eight samples was then used to adjust the participant DNA measurements to obtain the final T/S ratios in each batch. The coefficient of variability for the eight control samples across all batches was 6%. The T/S ratio for each participant was measured in duplicate. When the duplicate T/S value and the initial value varied by more than 7%, the sample was run for a third time, and the two closest values were used. Approximately 15% of samples required such assay in triplicate. Using this method, in this study the inter-assay coefficient of variability for telomere length measurement was 3.7% (equivalent to 0.20 kilobases with respect to the baseline mean). The intra-assay coefficient of variability was 2.5% (equivalent to 0.13 kilobases with respect to the baseline mean).

To determine the conversion factor for the calculation of approximate telomere length in base-pairs (mean size of terminal restriction fragments, TRF) from the T/S ratio, the above method was used to determine the T/S ratios, relative to the same reference DNA, for a set of genomic DNA samples from the human fibroblast primary cell line IMR90 at different population doublings, as well as with the telomerase protein subunit gene (hTERT) transfected via a lentiviral vector construct to elongate the bulk telomere population. For each of these DNA samples the mean TRF length was determined using Southern blot analysis, and the slope of the plot of mean TRF length versus T/S for these samples served as the conversion factor for calculation of telomere length in base pairs from the T/S ratio. The equation for conversion from T/S ratio to base pairs for this study was base pairs = 3274+2413*(T/S). Measurement of leukocyte telomere length was performed in a blinded fashion by Dr. Jue Lin without knowledge of the clinical data.

### Exercise capacity

Participants were instructed to fast for at least 4 hours before exercise, except for taking their usual medications. All subjects completed a graded exercise treadmill test according to a standard Bruce protocol, under the direct supervision of a cardiology technician and fellow. To achieve maximum heart rate, subjects unable to continue the standard Bruce protocol (for orthopedic or other reasons) were switched to slower settings on the treadmill and encouraged to exercise for as long as possible. Continuous electrocardiographic monitoring was performed throughout exercise. The only indications for stopping were those required for safety (systolic blood pressure >250 mm Hg, diastolic blood pressure >115 mm Hg, >1 mm ST elevation in leads other than V1 or aVR, >2 mm flat or down-sloping ST depression in 2 continuous leads, sustained ventricular tachycardia, new bundle-branch block, angina, or lightheadedness. Maximum exercise capacity was calculated, using standard equations based on workload (speed+grade) and duration of exercise, as total number of METs achieved at peak exercise (1 MET = 3.5 mL/kg/min of oxygen consumption). We evaluated exercise capacity both as a continuous variable and as a categorical variable, defined as low (<5 METS), moderate (5–7 METS) or high (>7 METS) exercise capacity [26]. Technicians performing the treadmill tests were blinded to the study goals.

### Physical activity

To assess self-reported physical activity, we asked, “Which of the following statements best describes how physically active you have been during the last month; that is, done activities such as 15–20 minutes of brisk walking, swimming, general conditioning, or recreational sports?” Participants chose from one of the following 6 categories: not at all active, a little active (1 to 2 times per month), fairly active (3 to 4 times per month), quite active (1 to 2 times per week), very active (3 to 4 times per week), or extremely active (5 or more times per week). We evaluated physical activity both as a 6-point continuous variable (corresponding to the above categories) and as a binary variable. For the binary variable, participants who reported that they were not at all or a little active were considered physically inactive; all other participants were considered physically active.

### Mortality

We conducted annual telephone follow-up interviews with participants (or their proxy). Death was determined by review of death certificates and coroner's reports.

### Other patient characteristics

Age, sex, race, education, smoking, alcohol use, and medical history were determined by self-report questionnaire. We measured height and weight and calculated body mass index. Participants brought all of their medication bottles to the study appointment, and study personnel recorded all current medications. Depressive symptoms were assessed using the 9-item Patient Health Questionnaire, and depression was defined as a score of ≥10 on this questionnaire [27]. Finally, all participants underwent resting echocardiography using an Acuson Sequoia ultrasound System (Mountain View, California). We obtained standard 2-dimensional views and performed planimetry with a computerized digitization system to determine resting left ventricular ejection fraction.

### Statistical Analyses

Baseline characteristics of participants were compared across categories of exercise capacity using ANCOVA for continuous and chi-squared tests for dichotomous variables. We used generalized linear models to calculate mean telomere length (T/S ratio) in patients with low, moderate and high exercise capacity. Models were adjusted for differences in patient characteristics associated with exercise capacity. Logistic regression models were used to evaluate the association between exercise capacity as a continuous variable and short telomere length as a binary variable (defined as having telomere length in the lowest quartile). Finally, we used Cox proportional hazards models to evaluate the association between exercise capacity and mortality, with and without adjustment for telomere length. We verified the proportional hazards assumption of these models. Analyses were performed using SAS Version 9.2 (Cary, North Carolina).

## Results

Of the 944 participants, 229 had low (<5 METS), 334 had moderate (5–7 METS) and 381 had high (>7 METS) exercise capacity (Table 1). Participants with greater exercise capacity were younger, more educated, and less likely to be current smokers. They had lower BMI and were less likely to be depressed. In addition, participants with higher exercise capacity had higher LVEF, and were less likely to have a history of hypertension, CHF, stroke, or diabetes. They were less likely to be taking renin-angiotensin system inhibitors, though more likely to be taking aspirin or statins. Participants with higher METS also had significantly greater self-reported physical activity (p<.001).

**Table 1:** Characteristics of 944 participants with coronary heart disease by exercise capacity (METS).

	Exercise Capacity			
	Low (<5 METS)	Moderate (5–7 METS)	High (>7 METS)	P value
	(n = 229)	(n = 334)	(n = 381)	
Age	71±11	68±10	63±10	<.001
Sex	184(80%)	272(81%)	330(87%)	0.07
White race	150(66%)	184(55%)	240(63%)	0.03
High school education	189(83%)	286(86%)	351(92%)	0.001
Current smoking	52(23%)	74(22%)	58(15%)	0.02
Regular alcohol use	59(26%)	92(28%)	126(33%)	0.10
Medical history				
Myocardial infarction	121(54%)	187(56%)	193(51%)	0.36
Hypertension	178(78%)	251(75%)	232(61%)	<.001
Heart failure	56(24%)	62(19%)	38(10%)	<.001
Stroke	48(21%)	52(16%)	28(7%)	<.001
Diabetes	76(33%)	91(27%)	69(18%)	<.001
≥weekly angina	50(22%)	64(19%)	58(15%)	0.11
Chronic lung disease	60(26%)	77(23%)	52(14%)	<.001
Body mass index	29.39±6.24	28.71±5.17	27.48±4.19	<.001
LV ejection fraction	0.61±0.10	0.62±0.10	0.63±0.09	0.009
Medication use				
Beta blocker	139(61%)	193(58%)	218(57%)	0.68
Aspirin (ASA)	169(74%)	258(77%)	314(82%)	0.03
ACE/ARB	135(59%)	166(50%)	183(48%)	0.03
Statin	127(55%)	230(69%)	258(68%)	0.002
Depressive symptoms	57(25%)	63(19%)	51(13%)	0.002
Physical inactivity	116(51%)	127(38%)	83(22%)	<.001

### Evaluation of mean telomere length (as a continuous variable)

**Figure 1:**
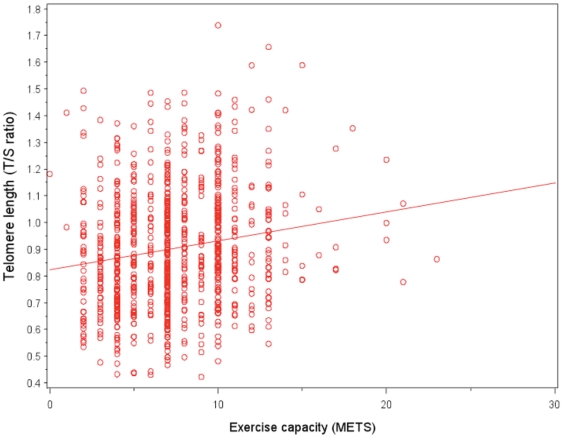
Scatterplot of exercise capacity and telomere length (r = 0.165, p<.001). Lower exercise capacity was significantly associated with shorter mean telomere length. METS = Metabolic Equivalent Tasks.

Lower exercise capacity was significantly associated with shorter mean telomere length (Figure 1, Table 2). Participants who had baseline METS <5 had a mean telomere length (T/S ratio) of 0.86 (5349 base pairs) while those with baseline METS >7 had a mean telomere length of 0.95 (5566 base pairs; p<.001). After adjusting for other differences in patient characteristics, including self-reported physical activity, those with METS <5 had a mean telomere length of 0.85 (5325 base pairs) compared with 0.92 (5494 base pairs) for those with METS >7 (p = 0.005).

**Table 2:** Mean (± SE) telomere length (T/S ratio) by exercise capacity.

	Exercise Capacity			
Model*	Low (<5 METS)	Moderate (5–7 METS)	High (>7 METS)	P value
	(n = 229)	(n = 334)	(n = 381)	
Unadjusted	0.86±0.21	0.88±0.21	0.95±0.23	<.001
Model 1	0.88±0.02	0.90±0.02	0.96±0.02	<.001
Model 2	0.86±0.02	0.87±0.02	0.94±0.02	<.001
Model 3	0.85±0.02	0.87±0.02	0.93±0.02	0.002
Model 4	0.85±0.02	0.86±0.02	0.92±0.03	0.005

*Model 1 = Adjusted for age, sex, race, education.

Model 2 = Model 1+hypertension, heart failure, stroke, diabetes, left ventricular (LV) ejection fraction, and chronic lung disease.

Model 3 = Model 2+angiotensin system inhibitor, statin, ASA.

Model 4 = Model 3+smoking, BMI, physical inactivity, and depressive symptoms.

Participants who reported being physically inactive had a mean telomere length (T/S ratio) of 0.88 (5397 base pairs), while those who reported being physically active had a mean telomere length of 0.91 (5470 base pairs; p = 0.02) (Table 3). After adjustment for age, sex, race, education, hypertension, heart failure, stroke, diabetes, LV ejection fraction, chronic lung disease, use of cardioprotective medications, smoking, body mass index and depressive symptoms, participants who reported they were physically inactive had a mean telomere length of 0.84 (5301 base pairs) compared with 0.87 (5373 base pairs) for self-reported physically active participants (p = 0.10).

**Table 3:** Mean (± standard error) telomere length (T/S ratio) by physical activity.

Model*	Physically inactive	Physically Active	P value
	(n = 326)	(n = 618)	
Unadjusted	0.88±0.01	0.91±0.01	0.02
Model 1	0.88±0.01	0.91±0.01	0.01
Model 2	0.85±0.02	0.89±0.02	0.03
Model 3	0.85±0.02	0.88±0.02	0.04
Model 4	0.84±0.02	0.87±0.02	0.10

*Model 1 = Adjusted for age, sex, race, education.

Model 2 = Model 1+hypertension, heart failure, stroke, diabetes, LV ejection fraction, and chronic lung disease.

Model 3 = Model 2+angiotensin system inhibitor, statin, ASA.

Model 4 = Model 3+smoking, BMI, and depressive symptoms.

### Evaluation of short telomere length (as a dichotomous variable)

**Figure 2:**
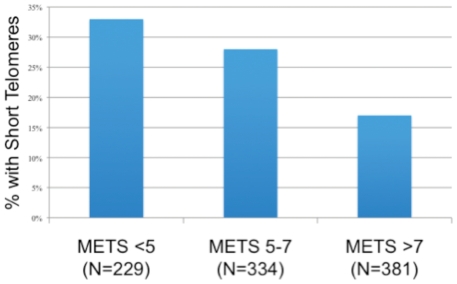
Proportion with Telomere Length in Lowest Quartile by Exercise Capacity (p<.001). Participants with lower exercise capacity were significantly more likely to have short telomere length (defined as being in the lowest quartile). METS = Metabolic Equivalent Tasks.

Self-reported physical inactivity was not independently associated with short telomere length (Table 4). However, participants with lower exercise capacity were significantly more likely to have short telomere length (defined as being in the lowest quartile) (p<.001) (Figure 2). For each standard deviation (SD) decrease in METS, participants had a 44% greater odds of having short telomeres (OR 1.44, 95% CI, 1.22–1.70; p<.001), and this association persisted after multivariable adjustment (OR 1.34, 95% CI, 1.07–1.67; p = 0.01) (Table 4). Compared with participants who had high exercise capacity (>7 METS), those with moderate exercise capacity (5–7 METS) had a 63% greater odds of having short telomere length (adjusted OR 1.63, 95% CI, 1.07–2.49; p = 0.02), and those with low exercise capacity (<5 METS) had a 94% greater odds of short telomere length (adjusted OR 1.94, 95% CI, 1.18–3.20; p = 0.009) (Table 5).

**Table 4:** Association of exercise capacity and physical activity (entered as continuous variables) with short telomere length, defined as having T/S ratio in the lowest quartile (<0.74 T/S or <5060 base pairs).

Model*	per SD (3.3-MET) decrease in exercise capacity		per 1-point decrease in physical activity score	
	Odds ratio (95% CI)	P value	Odds ratio (95% CI)	P value
Unadjusted	1.44 (1.22–1.70)	<.001	1.06 (0.97–1.15)	<.001
Model 1	1.38 (1.15–1.67)	<.001	1.07 (0.98–1.17)	0.15
Model 2	1.36 (1.12–1.66)	0.002	1.05 (0.96–1.15)	0.30
Model 3	1.35 (1.11–1.65)	0.003	1.04 (0.95–1.14)	0.38
Model 4	1.34 (1.07–1.67)	0.01	1.03 (0.94–1.13)	0.57

*Model 1 = Adjusted for age, sex, race, education.

Model 2 = Model 1+hypertension, heart failure, stroke, diabetes, LV ejection fraction, and chronic lung disease.

Model 3 = Model 2+angiotensin system inhibitor, statin, ASA.

Model 4 = Model 3+smoking, BMI, physical inactivity, and depressive symptoms.

**Table 5:** Association between exercise capacity and short telomere length, defined as having T/S ratio in the lowest quartile (<0.74 T/S or <5060 base pairs).

	Exercise Capacity				
	Low (<5 METS)		Moderate (5–7 METS)		High (>7 METS)
Model*	Odds ratio (95% CI)	P value	Odds ratio (95% CI)	P value	Odds ratio
Unadjusted	2.39 (1.60–3.55)	<.001	1.84 (1.27–2.67)	0.001	reference
Model 1	2.17 (1.41–3.33)	<.001	1.73 (1.17–2.55)	0.006	reference
Model 2	2.05 (1.30–3.23)	0.002	1.72 (1.14–2.57)	0.009	reference
Model 3	2.02 (1.28–3.21)	0.003	1.68 (1.12–2.52)	0.01	reference
Model 4	1.94 (1.18–3.20)	0.009	1.63 (1.07–2.49)	0.02	reference

*Model 1 = Adjusted for age, sex, race, education.

Model 2 = Model 1+hypertension, heart failure, stroke, diabetes, LV ejection fraction, and chronic lung disease.

Model 3 = Model 2+angiotensin system inhibitor, statin, ASA.

Model 4 = Model 3+smoking, BMI, physical inactivity, and depressive symptoms.

### Mortality

During an average of 6.27+/−2.11 years follow-up, each SD decrease in METS was associated with a two-fold increased risk of mortality (age-adjusted hazard ratio [HR] 2.09, 95% CI, 1.73–2.52;p<.001). This association was unchanged after adjustment for telomere length, sex, race, education, comorbid conditions, LV ejection fraction, use of cardioprotective medications, BMI, physical inactivity, and depression (HR 2.16, 95% CI, 1.70–2.75;p<.001). Likewise, each SD increase in baseline telomere length was associated with an 18% decreased risk of mortality (OR 0.82, 0.72–0.92), but this association was unchanged after adjustment for exercise capacity (OR 0.86, 95% CI, 0.76–0.98;p = 0.02).

## Discussion

We found a strong association between physical fitness and telomere length in 944 patients with CHD. After adjustment for other patient characteristics, including markers of cardiac disease severity and physical inactivity, participants with low exercise capacity (<5 METS) had a 94% greater odds of having short telomere length than those with high exercise capacity (>7 METS). In addition, participants with low exercise capacity had shorter mean telomere length than those with high exercise capacity (T/S ratio: 0.85 vs. 0.92, p = 0.005). This is equivalent to a difference of 169 base pairs (5325 vs. 5494 base pairs). Given that telomeres in this population decrease at an average rate of approximately 42 base pairs/year [28], this can be viewed as equivalent to a 4 year age difference between those with low versus high physical fitness.

Our study demonstrates for the first time a strong relationship between physical fitness and telomere length in a large sample of patients with existing CHD, after controlling for many other patient characteristics, such as severity of CHD. We extend the findings of previous studies on cardiovascular disease and telomere length [5], [6], [7], [8], [9], [10], [11], [12], [13], [14], [15], [16] by demonstrating that telomere length varies with fitness level within a population with known cardiovascular disease. The linkage between telomere length and an objective measure of fitness, rather than a self-reported one, is significant because it isolates aerobic fitness, rather than factors that tend to co-vary with greater self-reported physical activity, as being associated with longer telomeres. Many hard-to-measure factors that affect telomere length, such as nutrition and social stressors [29], [30], [31], [32], can differ between those who report exercise and those who do not, potentially limiting the conclusions that can be drawn from self-reported exercise [33], [34]. This may explain why previous studies examining the relationship between self-reported physical activity and telomere length have yielded mixed results [18], [19], [20], [35], [36], [37].

The causal direction of the association between fitness and telomere length cannot be determined by our analyses. Short telomeres could reduce exercise capacity by decreasing the function of the cardiovascular system, an idea supported by the fact that functional telomeres are required for viability of cardiovascular cells in-vitro and that deficient telomeres have been shown to cause cardiovascular disease in mice [38]. Alternatively, both low exercise capacity and short telomeres could result from common genetic or environmental factors. A third alternative is that physical inactivity could both reduce exercise capacity and shorten telomeres.

Several studies have suggested mechanisms by which physical inactivity may lead to shorter telomere length. The strongest evidence comes from research by Werner et al. showing that inactivity in mice alters the protein complexes that regulate leukocyte telomere length and structure. In this study, mice were randomized to running or no running wheel conditions for 3 weeks. The sedentary mice then showed lower telomerase activity, lower expression of telomerase reverse transcriptase (TERT), and lower telomere-stabilizing telomere repeat binding factor TRF2 compared with the aerobically conditioned mice [18], [39]. A subsequent observational study in humans found that sedentary individuals had decreased leukocyte telomerase and telomere-stabilizing protein compared to individuals with long-term endurance training [18]. Other research has suggested indirect mechanisms by which physical inactivity may lead to shorter telomeres. Moderate exercise has been shown to increase anti-oxidant capacity [40], [41], [42], and human studies have shown that higher oxidative stress levels lead to accelerated telomere shortening in leukocytes [13], [43]. Furthermore, in-vitro studies have demonstrated that oxidative stress increases telomere attrition [44], [45], [46] and decreases telomerase activity [47], [48] in numerous cell types [45], [47], [48], [49]. Yet another mechanism is that exercise may upregulate anti-inflammatory processes [40], [50], and increased inflammation may then contribute to telomere attrition [51], [52], [53].

Previous studies have shown that both exercise capacity and telomere length are powerful independent predictors of mortality among men with cardiovascular disease [17], [54]. Given the strong association between exercise and telomere length observed in our study, we investigated whether shorter telomere length mediated the association between exercise capacity and mortality. We found, however, that adjusting for telomere length had very little effect on the strong relationship between exercise capacity and mortality. These results suggest that telomere length does not contribute to the association between exercise capacity and mortality in patients with cardiovascular disease.

Among the strengths of the present study is the characterization of numerous clinical, biological, and psychosocial covariates that enable us to exclude possible confounders. However, our study has several limitations that should be considered in the interpretation of our results. First, the association reported in this study is cross-sectional, so no conclusions can be reached regarding causality. Next, no genetic polymorphisms were analyzed in our study, but genetics are known to impact telomere length in people with coronary artery disease [55]. Another limitation of our study is that most participants were urban, elderly men, and therefore the results may not generalize to other populations. Finally, our study population consisted entirely of people with stable coronary heart disease, and the results may not apply to either healthy individuals or those immediately post-myocardial infarction.

In summary, our study shows a strong relationship between exercise capacity and telomere length in a population of patients with stable coronary heart disease. The association between self-reported physical activity and telomere length became non-significant after multivariable adjustment. Whether poor physical fitness leads to shorter telomeres, or vice versa, and whether common genetic or other factors may reduce both telomere length and exercise capacity, deserve further study.
